# Genetic Influences on Outcomes of Psychotherapy in Borderline Personality Disorder: A Narrative Review of Implications for Personalized Treatment

**DOI:** 10.7759/cureus.43702

**Published:** 2023-08-18

**Authors:** Danya Ansari, Mohit Lakkimsetti, Kehinde T Olaleye, Jaskomal Veer K Bhullar, Rida Z Shah, Abimbola E Arisoyin, Huzaifa Nadeem, Sofia C Sacal Slovik, Fatima Z Habib, Zain U Abdin, Muhammad Zia ul Haq

**Affiliations:** 1 Psychiatry, Islamabad Medical and Dental College, Islamabad, PAK; 2 Internal Medicine, Mamata Medical College, Khammam, IND; 3 Neurological Sciences, AdventHealth Tampa, Tampa, USA; 4 Psychiatry, Government Medical College, Patiala, Patiala, IND; 5 Psychiatry and Behavioral Sciences, Dow University of Health Sciences, Karachi, PAK; 6 Internal Medicine, College of Medicine, University of Lagos, Lagos, NGA; 7 Psychiatry, Combined Military Hospital (CMH) Lahore Medical College, Lahore, PAK; 8 Neuroscience, Boston University School of Medicine, Boston, USA; 9 Programs & Research, SHINE Humanity, Karachi, PAK; 10 Medicine, District Head Quarter Hospital, Faisalabad, PAK; 11 Epidemiology and Public Health, Emory University Rollins School of Public Health, Atlanta, USA; 12 Noncommunicable Diseases and Mental Health, World Health Organization, Cairo, EGY

**Keywords:** mentalization-based therapy, dialectical behavior therapy, gene-environment interactions, dna methylation, epigenetics, psychotherapy, personalized therapy, treatment outcomes, genetic factors, borderline personality disorder

## Abstract

Borderline personality disorder (BPD) manifests as instability in mood, relationships, self-image, and behavior, representing a challenging mental health issue. This review scrutinizes genetic factors influencing BPD and the corresponding treatment outcomes. The primary objective of this narrative review is to illuminate the association between genetic factors and BPD treatment outcomes, discussing the potential of genetic testing for personalized therapy. The review is derived from observational and experimental studies on BPD, genetic factors, and psychotherapy from 2000 to 2023, sourced primarily through PubMed. Reviews and meta-analyses were excluded. Our review suggests that genetic factors account for 40-60% of BPD variation, with significant roles played by epigenetic alterations like DNA methylation and microRNAs, particularly in the context of childhood trauma. Gene-environment interactions are also vital for BPD's development. Treatments such as dialectical behavior therapy, mentalization-based therapy, and schema therapy have shown efficacy, with success variability possibly linked to genetic factors. However, existing research is constrained by recall bias, diverse methodologies, and limited sample sizes. Future research necessitates long-term follow-up, diverse populations, and controlled variables to enhance our comprehension of BPD treatment outcomes' genetic foundations. The review underlines the promise of personalized medicine in BPD treatment, driven by genetic insights.

## Introduction and background

Borderline personality disorder (BPD) is a serious mental illness that substantially impacts a person’s capacity to regulate their emotions. BPD presents with multitudinous symptoms such as an extreme fear of abandonment, a pattern of unstable and violent relationships, emotional lability, impulsivity, frequent self-destructive and suicidal behavior, short-lived stress-related paranoid thoughts, and identity issues.

A study found that approximately 2.7% of the U.S. population meets the criteria for BPD [1]. This means that about one in every 37 people may have BPD. However, the prevalence of BPD is not uniform across different groups of people. Some factors that may influence the likelihood of having BPD include gender, age, income, marital status, and race/ethnicity.

The prevalence is slightly higher in women (3.0%) compared to men (2.4%) and more prevalent in those with a lower income, under 30 years old, and those who are separated, divorced, or widowed [1]. Racial and ethnic differences in BPD prevalence are also eminent, with Native Americans and African Americans having higher rates of the disorder on average than Whites and Hispanics, while Asians have lower rates [1]. These variations may be influenced by a combination of genetic predispositions and cultural factors, such as family dynamics, social norms, values, beliefs, and coping styles.

BPD often co-occurs with other psychiatric disorders, such as anxiety disorders (84.8% of individuals), mood disorders (82.7%), and substance use disorders (78.2%) [1]. Psychiatric comorbidities like depressive disorders, bipolar disorder, anxiety disorders, and sleep disorders are more prevalent in female BPD cases, whereas substance use disorder and mental retardation are more prevalent in male BPD cases [2]. Furthermore, maximum cases with BPD have two or additional psychiatric comorbidities [2]. Medical comorbidities including arteriosclerosis, hypertension, hepatic diseases, cardiovascular disease, gastrointestinal disease, arthritis, and venereal diseases are also significantly associated with BPD [3]. The development of BPD is believed to be dependent on both genetic and environmental factors, such as childhood trauma, that can affect the hypothalamic-pituitary-adrenal axis, neurotransmission process, endogenous opioid system, and neuroplasticity [4]. These environmental factors act through epigenetic mechanisms like DNA methylation to negatively impact brain development [4].

While childhood trauma, physical and sexual abuse, and failed marriages are known to contribute to BPD's early emergence and severity, both genetic predispositions and environmental factors play a synergistic role in the pathogenesis of the disorder [5,6]. Taking genetics into account, some ethnicities are more prone to developing BPD, and it is known as one of the most inheritable psychiatric diseases [7]. Numerous genetic links with BPD have been identified through comprehensive meta-analyses, notably the L-type calcium channel subunit gene known as CACNA1C and the cell surface receptor protein named ODZ4 [8]. Given the significant role of genetics in BPD, emerging genetic testing offers an exciting opportunity to deepen our understanding of this complex disorder, potentially revolutionizing its treatment.

The treatment of BPD generally involves a comprehensive approach that combines psychotherapies, drug administration, and support systems [9]. Psychotherapies play a vital part in the treatment of BPD [9]. Some of the most common approaches include dialectical behavior therapy (DBT) which targets self-destructive conduct and interpersonal skills, mentalization-based therapy (MBT) which targets mental consciousness to abate symptoms, transference-focused psychotherapy (TFP), schema therapy, and acceptance and commitment therapy (ACT) that focus on ameliorating functioning and promote stability. Pharmacotherapy like selective serotonin reuptake inhibitors (SSRIs), antipsychotics, and anticonvulsants are used to treat specific symptoms.

Early intervention and management can improve the morbidity and mortality of this disorder. Increased suicide risk, often associated with impulsivity seen in BPD, contributes to higher mortality rates at younger ages. As patients become older, impulsivity might decrease, but other symptoms, such as emptiness, persist [10]. Understanding the importance of early prevention is crucial for improvement in patients. A large cohort study that began a few years ago has been instrumental in shaping the recognition and management of the disorder [11]. But studies including even larger populations can increase awareness and serve as guidance to providers to establish evidence-based practices. Investigation of gene-environment interactions may also orient therapeutic advancements and widen the dimensions of pharmacological and psychological management. It has been proven that environmental factors, like the experience of childhood abuse, play a role in BPD patients [12] Further knowledge of these factors and their interactions will help pave the way for advancements in the prevention, severity reduction, and overall management of the disorder.

The primary objective of this narrative review was to elucidate the complex interplay between genetic factors and psychotherapy outcomes in BPD. This involved comprehensively examining the existing literature to delineate the genetic underpinnings of BPD and how these may influence the efficacy of different psychotherapy modalities utilized for treatment. A secondary aim was to explore the potential role of genetic testing in guiding personalized treatment decisions for patients with BPD. By critically assessing the current state of knowledge in this field, we aimed to highlight areas that require further investigation and discuss the potential implications for clinical practice.

Literature search strategy

Database and Search Engines

We performed a comprehensive literature search of the PubMed database. Additionally, further relevant articles were identified through chain citations from the articles derived from the initial search.

Keywords and Search Terms

Our search strategy was developed using a combination of Medical Subject Headings (MeSH) and free text terms related to BPD, psychotherapy, and genetics. Specific search terms included “Borderline Personality Disorder”, “Psychotherapy”, “Genetics”, “Genetic Variants”, "Pharmacogenetics”, “Treatment Outcomes”, “Genome-wide Association Studies (GWAS)”, and "Gene-environment interaction" and related phrases. We constructed a comprehensive search matrix to ensure the inclusion of all relevant combinations of these keywords.

Inclusion and Exclusion Criteria

We included original research articles published within the past 10 years (2014-2023) that were written in English, with full-text availability, and primarily focused on the intersection of psychotherapy, BPD, and genetics. We excluded studies not primarily focused on these topics. Review articles, meta-analyses, and studies with significant methodological flaws were also not included in our review.

Timeframe

The literature search was restricted to studies published within the last 24 years (2000-2023) to ensure the inclusion of the most current research.

Quality assessment and data extraction

The quality of eligible studies was assessed using the Preferred Reporting Items for Systematic Reviews and Meta-Analyses (PRISMA) checklist. We evaluated the methodology, sample size, statistical analyses, and reported outcomes of each study. Furthermore, we also assessed potential biases. Key data such as study design, sample size, genetic variants studied, types of psychotherapy implemented, outcomes measured, and significant findings were extracted. Differences in data extraction were addressed by deliberation, and if required, the input of an additional reviewer was sought.

Key terms and concepts

Psychotherapy was defined as a therapeutic approach that uses dialogue and patient-therapist interaction to manage mental health disorders and improve psychological well-being.

Genetics delves into the intricacies of genes and inheritance patterns in living beings. Regarding BPD, this means exploring the impact of particular genes and their mutations on the onset, manifestations, and therapeutic reactions of the disorder.

Pharmacogenetics is the exploration of how one's genetic composition determines their drug response. This field merges the concepts of pharmacology, which pertains to drug science, and genomics, which focuses on genes and their roles, aiming to design medications that are both effective and safe, tuned to an individual's specific genetic makeup.

Precision medicine, sometimes referred to as tailored medicine, is a medical strategy that customizes treatments and interventions to fit the specific needs of each patient. This method frequently incorporates diagnostic evaluations to choose treatments that align with the patient's genetic makeup.

**Figure 1:**
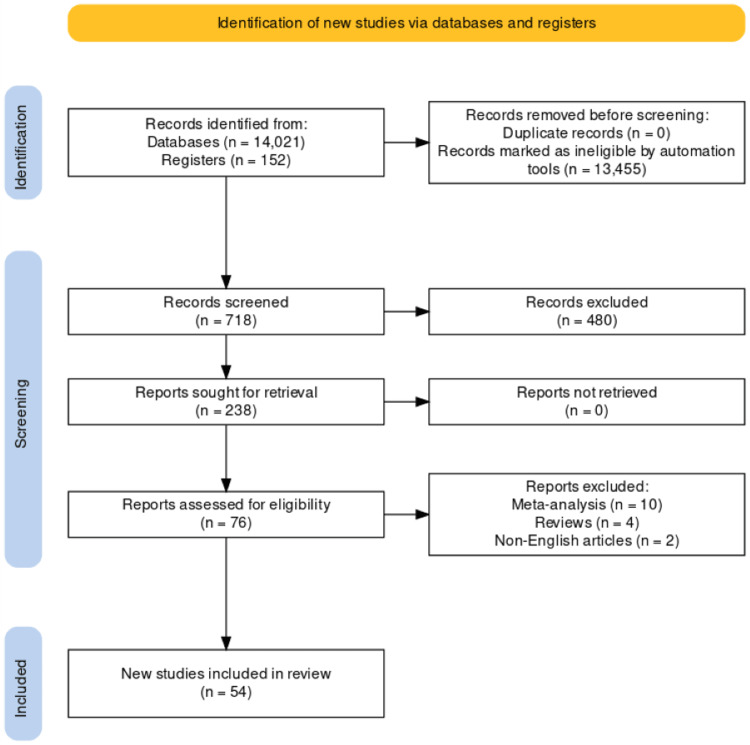
PRISMA flowchart illustrating the process of study selection from identification to inclusion PRISMA: Preferred Reporting Items for Systematic Reviews and Meta-Analyses

From our comprehensive search strategy and defined criteria, we initially identified a total of 14,173 studies (Figure 1).

After applying the inclusion and exclusion criteria, 718 studies were deemed eligible for review. Among these, a final set of 54 studies were included in the narrative review after a thorough evaluation of their quality and relevance.

## Review

Overview of genetic factors in BPD

Heritability of BPD

BPD is a valid diagnostic category with a strong genetic basis and a variety of effective psychotherapeutic treatments. Genetic components significantly contribute to BPD susceptibility, underscoring its familial trends [13]. Twin studies from different countries (The Netherlands, Belgium, and Australia) have consistently estimated that about 40% to 60% of the variation in BPD is due to genetic factors [14,15]. A large-scale study confirmed that relatives of BPD patients were more likely to have BPD than those without a family history of the disorder. A study estimated that 46% (95% CI = 39-53) of the variation in clinically diagnosed BPD was due to genetic factors [13].

According to the National Health Service (NHS), one of the main causes of BPD is genetic factors [16]. Furthermore, the NHS indicates that if one twin manifests BPD symptoms, the likelihood of the other twin developing BPD stands at 67%.

Epigenetic Factors Associated With BPD 

Genome-wide association studies (GWAS) scrutinize the entire genetic landscape to pinpoint both frequent genetic variations, like single nucleotide polymorphisms (SNPs), and rarer, yet consequential variants. This technique is a fast and cost-effective way to genotype individuals [15]. SNPs represent most of the genetic variation among individuals. This methodology offers an economical, rapid genotyping mechanism. Before such advancements, research on psychiatric disorders was constrained to select genes, often hindered by small participant numbers and gaps in understanding the disease's genetic architecture. However, there are few studies on the epigenetic changes that occur in BPD patients, even though these mechanisms are widely studied in relation to diseases linked to mental stress. A recent study has suggested that exposure to traumatic life events early in life can cause changes in the neurobiological system and brain morphology and emphasized the identification of biomarkers that may be involved in developing BPD [17]. Understanding the altered molecular and epigenetic pathways in BPD patients and how they are affected by childhood trauma may help to identify at-risk individuals and prevent or delay the onset of the condition in the future [17].

DNA Methylation 

As defined by the National Genome Research Institute, 'methylation is a chemical modification that occurs in DNA and other molecules and can be retained as cells divide to produce new cells'. Here, methyl group tags annex to DNA regions, governing gene activation or suppression. Research has demonstrated that epigenetic changes like DNA methylation may influence the interplay between genes and the environment and affect neurodevelopment in the context of BPD [18].

One of the ways this gene-environment interplay can be studied is by examining DNA methylation in individuals diagnosed with BPD. A study first using the epigenome-wide study on patients diagnosed with BPD (N=96) and controls (N = 44) and then using EpiTYPER assays (N = 293 BPD patients and N = 114 controls) to confirm, revealed that compared to controls who were healthy, BPD patients had considerably lower levels of methylation at cytosine-phosphate-guanine (CpG) sites [17]. DNA methylation sites termed CpG islands occur in promoters and are known to regulate gene expression through transcription silencing the associated gene. The EpiTYPER test is a high-resolution scan of chosen regions carried out for DNA methylation detection and quantitative analysis. Thus, it permits a more thorough investigation of individual CpGs and their surroundings and can be used to validate CpGs found by genome-wide methods in addition to candidate gene methylation studies [19].

A gene set enrichment research study indicates that genes controlling estrogen synthesis, neurogenesis, and cellular differentiation undergo the most epigenetic changes [20]. This suggests that differences in how BPD manifests between genders might originate from epigenetic modifications in genes on the X chromosome and those involved in estrogen production [21].

Moreover, four specific genes (POU5F1, GGT6, TNFRSF13C, and FAM113B), which are not on the X chromosome, display notable differences between BPD individuals with a history of childhood trauma and those without [22]. Other genes such as HTR2A, MAOA, MAOB, COMT [23], and NR3C1 [24] have displayed changed methylation patterns in BPD patients.

MicroRNAs 

Research indicates that DNA methylation is not the sole mechanism linked with childhood trauma and its potential relation to BPD onset [21,22]. MicroRNAs (miRNAs), small non-coding RNAs that control gene expression by obstructing mRNA translation, have also been implicated. Within BPD, there is a notable connection between miRNAs and the intensity of childhood abuse, suggesting their potential role in the disorder's development [25]. miRNAs can modify epigenetic regulator expressions, which indirectly affects overall gene expression [26]. 

One research pinpointed a specific CpG site, cg04927004, near the gene for miRNA (miR124-3). This site is strongly linked with the severity of childhood trauma and BPD, hinting at its role in the progression from early traumatic experiences to adult BPD. Additionally, this research identified another gene, miR137, related to childhood trauma, which targets NT5DC2-a gene related to both schizophrenia [25] and BPD.

Gene-Environment Interactions

Linehan's biosocial model offers insight into the origins and behaviors of BPD. This model posits that BPD arises from the interplay of biological predispositions and environmental influences [25,27]. Innate tendencies, like impulsivity and heightened emotional sensitivity, predispose individuals to strong, often negative emotions, increasing BPD susceptibility [28].

On the other hand, environmental factors include invalidating environments, which are characterized by a lack of acceptance, support, and understanding of the individual’s emotional needs and expressions [29]. An example could be a household where a child's emotional expressions are frequently dismissed or criticized. The biosocial model also proposes that these factors influence each other in a transactional manner, such that biological vulnerabilities are exacerbated by environmental stressors, and vice versa, leading to a vicious cycle of emotional and behavioral dysregulation [25].

Biological predispositions primarily lead to two outcomes: impulsivity and emotional sensitivity. Those developing BPD typically experience adverse family conditions. One study delved into the interplay of genetic and environmental factors by contrasting familial risk factors' effects, encompassing parental mental health issues, borderline tendencies, ineffective parenting strategies, and marital strife, on BPD traits in biologically related and adopted families [27].

This study discovered that only in biological offspring did the father's BPD characteristics significantly forecast BPD traits during childhood, hinting at genetic transmission. Manifestations of parental behavioral disorders, adult antisocial tendencies, and dependencies on substances like nicotine, alcohol, and illicit drugs were also major indicators of BPD traits in offspring. However, irrespective of the adoption status, traits like maternal BPD characteristics, conflict between parents, neglect, and lack of involvement were consistent predictors, pointing toward environmental influences [30].

Psychotherapeutic approaches to BPD

Dialectical Behavior Therapy

DBT is an evidence-based treatment for BPD. Marsha Linehan, credited for DBT's inception in 1993, described BPD as a dialectical challenge requiring extensive therapy for sustainable improvement [32]. She described a series of cognitive behavioral therapies (CBT) specifically tailored to BPD involving validation, problem-solving, managing contingencies, and cognitive modification while emphasizing communication between patient and therapist [31]. These therapies laid the foundation of DBT.

Although DBT demonstrates effectiveness, patients with BPD often need extended periods for marked improvement. The duration and setting of DBT (inpatient or outpatient) are adapted based on the specific symptoms and clinical requirements of individual cases.

An outpatient-based study in Switzerland involved 127 BPD patients. After undergoing three weeks of intensive DBT-including both group and personal sessions, alongside skills and mindfulness training, participants reported significant reductions in feelings of depression and hopelessness [32].

To recruit patients for trials, the DSM-IV structured interview clinical criteria are used to assess the patients' BPD diagnosis. A study by Barnicot et al. recruited 90 patients in a non-randomized comparison between DBT and MBT [33]. The outcomes included incidents of self-harm and emotional dysregulation. Other baseline measures used were Axis I to detect criterion A traumatic event or Mini International Neuropsychiatric Interview (MINI) to assess major depression/ substance abuse. The use of multiple standardized questionnaires helped to identify consistent treatment outcomes despite being a multi-center study. Over the course of a year, participants underwent individual therapy, group training, phone coaching, and team consultations across five UK-based NHS trusts. Results showed that 58 participants who received DBT and associated follow-up experienced lower incidences of self-harm. The authors of this study then compared DBT against MBT, which we have further explored in the section below. The study adjusted for confounding due to treatment dropout and use of emergency services [33].

Mentalization-Based Therapy

Mentalization, a type of social cognition, is influenced by early social experiences. It encompasses an individual's ability to interpret their own and others' mental states [34]. Impaired mentalization, often linked to early relationship experiences and trauma, is a central BPD feature, complicating interpersonal relationships. MBT primarily addresses this deficit, especially under stressful social interactions [35].

MBT aims to enhance patients' functioning in regular social interactions and improve their quality of life by addressing a core weakness in BPD patients [34]. Initially designed to be utilized for around 18 months, MBT includes weekly individual and group sessions as well as any additional medical care if needed. It was initially tested in patients with BPD in a partial hospital setting.

This therapy is particularly beneficial for individuals struggling with intense emotions and self-destructive behaviors, interpersonal challenges, and difficulty predicting others' reactions. Results have shown diminished BPD symptoms, reduced concurrent mental health issues, and enhanced life quality.

Children, adolescents, and families that experience psychological problems might benefit from MBT. Recent research highlighted that an 18-month MBT regimen yielded significant improvements for BPD patients, with those on MBT experiencing faster reductions in clinically relevant issues, such as suicide attempts and hospitalizations, compared to a structured clinical management outpatient methodology [36]. The uniqueness of MBT lies in its holistic integration of various components, with a dedicated focus on augmenting mentalization capacities, differentiating it from other psychotherapies [37].

MBT for BPD was the initial program of its kind to be established. Its novelty lay not in the individual components, but in the unique integration of these elements, as well as the persistently determined approach encouraging the therapist to boost mentalizing capabilities. This particular emphasis on enhancing mentalizing is the key differentiator that sets mentalizing therapies apart from other forms of psychotherapy [37].

The overarching objective is to help patients introspect their perceptions and emotions about themselves and others, comprehend their behavioral implications, and recognize how misinterpretations may lead to maladaptive actions [37].

Schema Therapy

Schema therapy is a cognitive schema-based integrative psychotherapy that identifies and transforms faulty schemas and modes using cognitive, experiential, and behavioral means [38]. It was designed specifically for patients with personality disorders. Schemas are patterns or themes that a person develops in childhood about themselves and their relationships with others. Developed especially for personality disorders, schemas represent consistent patterns or themes individuals develop during their early years. Schema therapy is a style of integrative psychotherapy that incorporates CBT, Gestalt Therapy, Attachment Theory, and Psychodynamic perspectives [39]. 

Schema therapy is rooted in two foundational theories. Early maladaptive schemas (EMS) are deep-seated patterns that usually originate in childhood, stemming from discrepancies between a child's needs and their environment [39]. The schema mode model, meanwhile, postulates that BPD patients' rapid emotional shifts are due to simultaneous activation of various schemas. This model delves into the patient's current state, aligning with their schemas and coping mechanisms at a particular moment [39].

In BPD patients, four problematic modes are common, i.e., abandoned/abused child mode, angry/impulsive child mode, detached protector mode, and punitive parent mode. In the vulnerable child mode, patients typically grapple with intense emotions, seeking external support [39].

Transference-Focused Psychotherapy (TFP)

TFP, with its psychodynamic foundation, is particularly effective for enhancing reflective functioning in borderline patients. TFP aims to change how self- and other representations are played out in the present transference to lessen symptomatology and self-destructive behavior [20].

Comparing Efficacy and Effectiveness Across Therapies and Outcomes

Organized conversations that examine the long-term functioning of individuals with BPD enable us to identify and treat patients with confidence. DPSI-IV (demographic psychosocial inventory) is a semi-structured interview method based on DSM-IV BPD criteria used to assess the patient's treatment outcomes. Structured interviews are a valuable tool in diagnosing and treating individuals with BPD. These interviews provide a comprehensive understanding of the patient's long-term functioning, essential for accurate diagnoses and effective treatments. Using such a strategy on a large number of patients yields data acceptable for long-term treatment-outcome study. This self-reported questionnaire can provide information about psychological risk factors. It is pivotal to compare various therapeutic methods in BPD to create customized patient-centered treatment strategies.

Different trials have analyzed outcomes from various therapeutic techniques. For instance, a Netherlands-based trial with 200 BPD patients compared DBT and schema therapy. Primary outcomes focused on BPD symptoms, while secondary ones evaluated overall well-being and additional patient-reported symptoms. The study assessed the clinical and economic benefits of both therapies. While DBT participants reported fewer self-harm instances and reduced emotional dysregulation compared to MBT participants, there were no substantial differences in BPD manifestations or interpersonal issues [40].

Another research project was designed to compare the efficacy of MBT and DBT [33]. The study emphasized that regardless of the context it's provided in, the primary aim of MBT is to enhance the process of mentalization. Unlike DBT, an MBT therapist does not actively seek to alter behavior, provide insights, or engage in cognitive restructuring, which distinctly differentiates the two approaches. It would be inaccurate to assume that patients undergoing MBT lack understanding of deeper meanings or insight into their conditions. It is also incorrect to say that they do not experience any behavioral or cognitive changes during mentalizing therapy. Indeed, evidence points toward such transformations happening, but they appear more like secondary occurrences, instigated by a shift in mentalization [23]. The strongest empirical backing has been given to DBT and MBT therapies, based on clinical trials [41]. Despite their unique characteristics, these therapies share many similarities. Therefore, it could be advantageous to blend them to enhance their accessibility and potential benefits.

A recent randomized controlled trial explored the effectiveness of MBT in structured clinical management for individuals who have co-occurring BPD and antisocial personality disorder (ASPD) [42]. The study's findings demonstrated that MBT was particularly effective in mitigating symptoms such as anger, hostility, paranoia, and impulse control problems in patients with concurrent BPD and ASPD. In addition, the use of MBT was associated with a decrease in the instances of self-harm and suicide attempts. The treatment also led to improvements in general psychiatric symptoms, interpersonal problems, social adjustment, and overall patient functioning.

A recent research project analyzed the cost-effectiveness of two therapeutic approaches, TFP and schema therapy, in managing BPD. The goal was to determine the economic viability of each approach. The study measured the associated costs and evaluated health-related quality of life using the EQ-5D (EuroQol-5 dimension) questionnaire. The findings revealed that over a span of four years, schema therapy averaged $37,826 in costs, whereas TFP came in higher at $46,795 [41]. In terms of quality-adjusted life-years (QALYs), schema therapy recorded 2.15, slightly lower than TFP's 2.27. Both treatments were beneficial in enhancing life quality. Examining full recovery rates, the study highlighted that 52% of the participants in the schema therapy group achieved full recovery, while only 29% in the TFP group reached the same milestone.

Consequently, schema therapy boasted a better overall recovery success [41]. Summarizing the findings, schema therapy emerges as a more economically favorable choice for BPD treatment compared to TFP, with a cost-saving of about $90,457 per QALY reduction [41].

Influence of genetic factors on psychotherapy response

In a 2013 study, Perroud and his team explored methylation patterns in BDNF (brain-derived neurotrophic factor) and its potentially reduced expression [43]. All subjects received four weeks of intense DBT (iDBT), with 115 patients diagnosed with BPD and 52 controls. This treatment approach is typically more structured and concentrated than standard DBT, with a stronger emphasis on leveraging the individuals' strengths, abilities, and future potential. The study found a decrease in methylation status in response to the iDBT when responders were compared to non-responders. Non-responders experienced an increase in DNA methylation [43]. The researchers also discovered a link between childhood trauma/maltreatment and BDNF DNA methylation. Depression severity, despair, and impulsivity were also significantly reduced with time.

A pilot study in 2022 conducted by Quevedo in 11 females with early trauma found stress reduction seen in peripheral FKBP5 response protein methylation in response to BPD psychotherapy. The results implied that psychotherapy affects epigenetics involved in BPD's development and psychotherapeutic response [44]. A study in 2017 led by Knoblich et al., involving 88 participants (comprising 37 females and seven males diagnosed with BPD, matched with 44 healthy controls), found elevated methylation levels in APBA3 and MCF2 among those responding to a 12-week DBT program, emphasizing the predictive power of genetic indicators in BPD treatment [45].

In essence, these studies underscore the intricate relationship between genetic factors, particularly DNA methylation, and psychotherapy's efficacy in BPD management.

Limitations and Inconsistencies in the Current Literature

The symptoms of BPD and long-term trial outcomes are primarily measured through patient self-reporting in interviews. This approach is susceptible to recall bias, which impedes retrospective comparisons and necessitates longer follow-up trials. Moreover, the variability in methodologies across different studies creates challenges in comparing results, highlighting the need for standardized structured interviews or questionnaires.

A further complicating factor is the variation in definitions of a 'response' to treatment, making it difficult to derive definitive conclusions. There are other confounding variables to consider as well, such as small sample sizes, concurrent use of alternative medications and therapies, patient noncompliance or dropout, and use of emergency services during treatment. Another vital consideration includes the quality of therapist training and the accuracy of the BPD diagnosis, especially given the frequent comorbidities associated with BPD, such as substance use disorder and PTSD.

Moreover, another crucial oversight in the current literature is the potential impact of cultural epigenetic and trauma factors on treatment response. Cultural influences can shape how individuals perceive and react to treatment, while trauma, especially if experienced across generations, can have epigenetic implications that might affect treatment outcomes. Recognizing and addressing these factors can offer a more nuanced understanding of BPD and its treatment dynamics.

Many studies are currently restricted in scope, focusing predominantly on females and running for a relatively short duration. Short-term trials, usually lasting 4-8 months, cannot fully capture the duration of treatment. Also, single-center studies may be constrained by the treatments that the particular center offers.

These limitations make it hard to isolate the effects of psychotherapy, as many BPD patients might also be using medication or undergoing other forms of therapy. To better define future care, we need expansive, longer-duration studies that embrace a diverse demographic, inclusive of various genders, mature age groups, pertinent genetic factors, and distinct BPD symptom clusters.

Implications of genetic testing for BPD treatment decisions

Potential Benefits

The potential for genetic testing to guide treatment choices for different illnesses, including BPD, is highlighted by the growing interest in personalized medicine. Clinicians may better tailor treatments for individual patients by identifying genetic variants linked to therapy responses.

Research supports this idea of customization. One study showed that patients undergoing CBT exhibited changes in the methylation of their DNA in their serotonin transporter gene [46]. This gene encodes the protein facilitating serotonin reuptake in neuron synapses. Another study found a correlation between variations in the BDNF gene and enhanced therapeutic responsiveness [43]. BDNF is a protein essential for the development, survival, and maintenance of brain neurons. Patients with specific variants of this gene showed more significant improvement in symptoms of impulsivity and suicidality.

In the same way, certain medications may affect some individuals more positively than others which can also help tailor the approach of genetic testing in the management of BPD. A randomized, controlled trial found that oxytocin receptor gene differences were linked to the increased or decreased sensitivity of a person to oxytocin [47], Solidifying the notion that implications of genetic testing may be of significance.

Combining pharmacotherapy and psychotherapy could also significantly impact treatment outcomes, as suggested by another study that found patients undergoing interpersonal psychotherapy and taking antidepressants achieved better results [48]. However, the number of studies correlating pharmacotherapy and psychotherapy to the same genetic factors remains sparse. A limited amount of research is currently available linking both types of treatments to any one gene.

Ethical Concerns in Genetic Testing

The implications of genetic testing not only bear significant potential, but also introduce a host of ethical concerns that impact individuals, healthcare providers, and society as a whole. Misuse or prejudice due to unsolicited access to genetic information remains a significant risk if not adequately managed.

At an individual level, concerns revolve around patient autonomy and privacy. Proper informed consent processes are vital, ensuring patients fully understand the potential risks and consequences of genetic testing and data management [49]. Failing to provide adequate informed consent undermines patient autonomy and trust in the healthcare system, potentially deterring participation in research studies and sharing of data.

Moreover, patients may face stigmatization upon detection of certain genes, affecting their psychological well-being and social relationships [50]. This issue is evidenced in a study on the stigma surrounding genetic testing for mental health disorders, such as schizophrenia, where patients encountered bias from medical professionals, family, and society, leading to concealment and reluctance to share genetic information [51]. This fear of discrimination can influence society broadly. It correlates with the fear of discrimination, due to genetics, which was evident in patients in a previous study [52]. The drawback to disclosing information to patients is that this might cause increased anxiety and undue stress in patients, affecting their daily life and decisions as they may be unable to completely grasp the implications of their results [48]. 

While the Genetic Information Nondiscrimination Act (GINA), enacted in the United States in 2008, prevents discrimination based on genetic information, similar protections may not exist worldwide. Consequently, understanding local laws and regulations around genetic data is crucial for both patients and practitioners. One trial reported initial patient apprehension about genetic testing due to data misuse but revealed more positive perceptions once patients understood the protective measures in place [52,53].

From a healthcare provider's perspective, effectively communicating complex genetic results while maintaining the patient's emotional well-being and comprehension presents an ethical challenge, impacting shared decision-making and personalized care. Furthermore, poor data management could impede the progress of genetic research.

Proactively addressing these issues will champion an ethical, patient-centric approach to genetic testing, fostering healthcare trust and maximizing its benefits.

**Figure 2:**
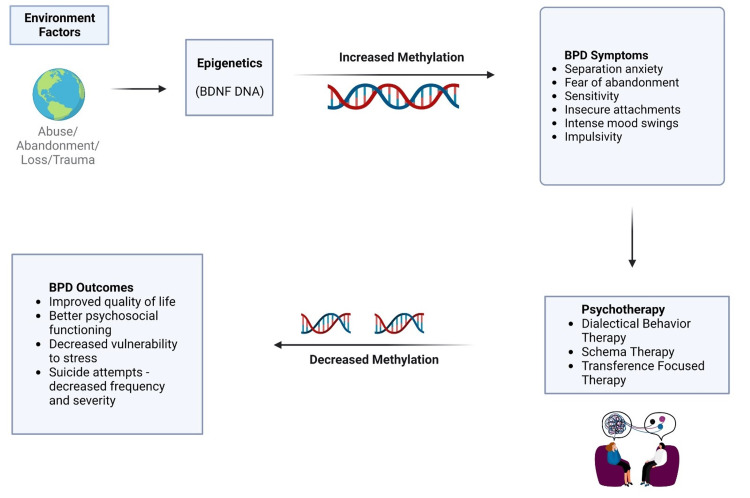
Interplay of environmental and epigenetic factors and psychotherapy on BPD outcomes and symptoms BDNF: Brain-derived neurotrophic factor; DNA: deoxyribonucleic acid; BPD: borderline personality disorder

As illustrated in Figure 2, increased methylation of BDNF DNA can exacerbate BPD symptoms, such as separation anxiety, fear of abandonment, sensitivity, insecure attachments, intense mood swings, and impulsivity. Conversely, decreased methylation can lead to improved psychosocial functioning, quality of life, and resilience against stress, while reducing the frequency and severity of suicide attempts.

Figure 2 also highlights the importance of psychotherapy, particularly DBT, in modulating these outcomes. DBT, a cognitive-behavioral treatment, has been shown to effectively treat chronically suicidal individuals and those with severe emotion dysregulation, including individuals diagnosed with BPD. The positive impacts of DBT on these patients are reflected in the decreased BPD symptom severity.

Simultaneously, the illustration acknowledges the intricate relationships between these components. For instance, environmental factors might influence epigenetic modifications, which in turn could affect response to psychotherapy. Understanding these dynamics is an ongoing research endeavor and is instrumental in optimizing therapeutic strategies for BPD. A selection of most important studies is summarized in a comprehensive table (Table 1).

**Table 1:** Comparative analysis of selected studies on treatment approaches for borderline personality disorder BPD: Borderline personality disorder; DBT: dialectical behavior therapy; MBT: mentalization based therapy; TFP:  transference-focused psychotherapy; ASPD: antisocial personality disorder; SFT: solution-focused therapy

Study Name	Outcome Measures	Key Findings	Limitations
Characteristics of BPD in a community sample: Comorbidity, treatment utilization, and general functioning [1]	Prevalence and demographic features of BPD	Racial and ethnic differences with higher rates in females, people in lower income brackets, people age<30, individuals who are divorced or separated.	Racial and ethnic differences in treatment utilization and general functioning no clear data
Comorbidity study of borderline personality disorder: applying association rule mining to the Taiwan national health insurance research database [2]	Physical comorbidities and psychiatric comorbidities	Increased frequency of both physical and mental comorbidities, except for congenital abnormalities.	Study was for a short duration; subjects differ from the general population and no structured interviews or standard diagnostic criteria were used.
Comorbidity and associated severity of BPD and physical health conditions in a nationally representative sample [3]	Association of BPD with physical health conditions	BPD has correlations with diseases such as arteriosclerosis, hypertension, liver-related disorders, cardiovascular issues, and arthritis. However, there is no direct link with diabetes, strokes, and obesity.	
Borderline personality disorder and childhood trauma: exploring the affected biological systems and mechanisms [4]	Biological system and environmental factors associated with BPD pathogenesis.	The vulnerability to develop BPD due to childhood trauma can be linked to modifications in the HPA axis, neurotransmission, the endogenous opioid mechanism, and neuroplasticity.	No clear role of genetic factors
The Lifetime Course of Borderline Personality Disorder [10]	Onset and progression of BPD throughout life, including childhood, adolescence, and adulthood	The onset of BPD typically occurs during the adolescent years. It isn't always a chronic condition, as many individuals exhibit lingering symptoms in their later years.	Limited research on BPD in older individuals
The McLean Study of Adult Development (MSAD): Overview and Implications of the First Six Years of Prospective Follow-Up [11]	First NIMH-funded prospective study of the course and outcome of BPD	Remissions are far more common, completed suicides are far rarer and there is improvement psychosocially over time.	
Trauma and Outcomes of Mentalization-Based Therapy for Individuals With Borderline Personality Disorder [12]	The frequency and repercussions of childhood trauma in BPD patients who participated in an RCT that contrasted MBT in day hospitals (MBT-DH) with intensive outpatient MBT (MBT-IOP).	The presence of childhood trauma didn't substantially influence the results of either MBT-DH or MBT-IOP. However, individuals with past emotional neglect experienced swifter symptom shifts in BPD under MBT-DH.	Bias (Recall and reporting) and small sample size.
A twin study of personality disorders [14]	The interplay of genetic factors and environmental conditions plays a role in the emergence of personality disorders.	Heritability of .60 for personalities disorder and .69 for BPD.	Moderate sample size and low prevalence of specific disorders
Potential epigenetic mechanisms in psychotherapy: a pilot study on DNA methylation and mentalization change in borderline personality disorder [18]	Early trauma, BPD symptoms, depression symptoms, emotional regulation, mentalization, and decrease in FKBP5 methylation.	Epigenetic modifications related to therapeutic interventions were exclusively identified in patients who experienced trauma during their early years.	Limited sample size with physical and socioeconomic factors not controlled.
Familial factors and the risk of borderline personality pathology: genetic and environmental transmission [27]	Parental attributes, behaviors, and mental health disorders were assessed through structured clinical dialogues. The connection between family-related risk factors and BPD traits in children was explored using multi-tiered regression analyses.	Genetic factors influence symptoms like a parent's conduct disorder, antisocial tendencies, dependencies on nicotine, alcohol, and illicit drugs, and paternal BPD characteristics. Meanwhile, environmental factors play a role in maternal BPD traits, conflicts between parents, and reduced parental involvement with the child.	
Psychotherapy of borderline personality disorder [30]	Self-mutilation and suicide attempts	All four psychotherapy methods showed reduced outcomes.	All psychotherapies are long and intensive in clinical duration and other than DBT, therapies are not well studied.
Intensive Dialectical Behavior Therapy for Outpatients With Borderline Personality Disorder Who Are in Crisis [32]	Depression and hopelessness	Reduced incidence of undesired outcomes	A short study period that did not control for many factors and suicidal behavior was not measured.
Dialectical behaviour therapy v. mentalisation-based therapy for borderline personality disorder [33]	Events of self-inflicted injuries (modified IRR), emotional instability (modified β value), premature treatment cessation, resorting to emergency services, BPD manifestations, and issues in interpersonal relationships.	Compared to MBT, DBT displayed a more pronounced reduction in self-harming actions and emotional instability. However, the variations in premature treatment termination, emergency service utilization, BPD symptoms, or interpersonal relationship challenges between the two therapeutic approaches weren't considerable.	Non-randomized study design limits causal conclusions and long-term outcomes beyond 12 months were not assessed. Significant number of dropout patients
Mentalization based treatment for borderline personality disorder [34]	Improvement in mentalizing abilities, emotional regulation, impulse control, interpersonal functioning, and relationship outcomes.	Effectiveness of MBT for BPD and its delivery by mental health professionals with limited training. Limited additional train	Generalizability, long-term effectiveness, and challenges in implementing MBT in different mental health settings.
Treatment of personality disorder [37]	Effective management of personality disorders and BPD.	BPD psychological or psychosocial intervention as primary treatment, pharmacotherapy as adjunctive treatment.	Lack of research on the interaction between psychotherapies and drugs, and insufficient understanding of the manifestation of disordered personality and its underlying processes.
Implementation of outpatient schema therapy for borderline personality disorder: study design [38]	Before, during, and after therapy, a structured interview and self-assessments on BPD, overall life quality, and general psychiatric symptoms	Implementation of ST in regular mental healthcare practice and Extra assistance from the therapist outside of sessions if they require it due to a crisis or emotional need.	Research involved a multicenter, two-group, randomization study to examine the benefits of therapist phone help outside of office hours.
Schema therapy for borderline personality disorder: A qualitative study of patients’ perceptions [39]	Clinical features such as self-confidence, insight, control of emotions and cognitive flexibility	BPD recovery was associated with increased self-assurance, improved self-awareness, less self-blame, and more self-acceptance.	Small sample size
Towards optimal treatment selection for borderline personality disorder patients (BOOTS): a study protocol for a multicenter randomized clinical trial comparing schema therapy and dialectical behavior therapy [40]	Mechanisms of change with different therapies such as beliefs with schema, emotional regulation with DBT, genuineness, safety, and attachment	Prospective multicenter RCT idea to help tailor individual treatment for BPD.	Study not performed yet.
Out-patient psychotherapy for borderline personality disorder: Cost-effectiveness of schema-focused therapy v. transference-focused psychotherapy [41]	Costs per recovered patient according to BPDSI, quality-adjusted life years (QALYs)	When comparing SFT to TFP, 52% of SFT patients showed recovery, as opposed to 29% for TFP. Moreover, SFT proved to be more economical and efficacious than TFP.	Limited to just two treatments, did not control for disorder-related costs and no guidance on decision-making of which psychotherapy to use.
A randomized controlled trial of mentalization-based treatment versus structured clinical management for patients with comorbid borderline personality disorder and antisocial personality disorder [42]	The influence of MBT versus SCM on multiple symptoms in patients diagnosed with both BPD and ASPD was examined.	MBT was successful in diminishing symptoms like anger, impulsiveness, paranoia, and hostility in these patients. There were also noted reductions in instances of self-harm and suicide attempts, and improvements were seen in mood, psychiatric symptoms, social interactions, and overall social adaptation.	Short follow up with population being under representative of wider ASPD population and less power to demonstrate significant differences between the two groups.
Response to psychotherapy in borderline personality disorder and methylation status of the BDNF gene [43]	The study centered on the DNA methylation present in the BDNF gene and the significance of this epigenetic factor in psychotherapy.	A direct connection between childhood trauma and BDNF methylation was identified. Patients who positively responded to DBT exhibited a marked reduction in methylation as time progressed.	Study tested peripheral blood for changes in the brain – possibly not entirely accurate representation and used varied pharmacotherapies with short follow-up.
DNA methylation of APBA3 and MCF2 in borderline personality disorder: Potential biomarkers for response to psychotherapy [44]	Examination of DNA methylation at epigenetic locations: notably APBA3 and MCF2.	Therapy responders showed increased levels of methylation prior to treatment compared to no responders – this could indicate a prognostic value for these epigenetic markers.	Study had limited sample size with no control for comorbidities and no use of pharmacotherapy with DBT.
Serotonin transporter methylation and response to cognitive behavior therapy in children with anxiety disorders [45]	The study aimed to highlight variations in SERT methylation that correlated with reactions to exclusively psychological treatments.	Changes in DNA methylation throughout the therapy were linked with positive outcomes to CBT in pediatric anxiety disorder patients.	Small sample size and no DNA samples were taken to measure percentage DNA methylation at the follow-up time point, so no comparison of results could be done.
Genetic modulation of oxytocin sensitivity: a pharmacogenetic approach [46]	The research probed the influence of genetic alterations on susceptibility to externally supplied oxytocin.	A distinctive six-marker haplotype block that spanned the promoter region and intron 3 was found to be notably linked with oxytocin sensitivity measurements, elucidating why some individuals react more intensely to oxytocin in terms of emotion recognition.	The study was limited to male participants only.
Adaptation of Interpersonal Psychotherapy to Borderline Personality Disorder: A Comparison of Combined Therapy and Single Pharmacotherapy [47]	The study assessed if an amalgamated treatment approach, utilizing a revised form of interpersonal psychotherapy, retained an edge over antidepressants.	The amalgamated treatment that incorporated the modified IPT outperformed fluoxetine as a standalone, although the remission rates between the two showed no distinction.	Population has different clinical characteristics from patients treated in clinical practice and study did not include intention-to-treat analyses.
Attitudes of healthy volunteers to genetic testing in phase 1 clinical trials [48]	The research delved into the perspectives of healthy test subjects in initial phase studies regarding genetic confidentiality, incidental genetic results, and overall genetic privacy.	A significant proportion of these healthy individuals were inclined towards receiving incidental genetic discoveries, and a majority deemed genetic privacy and security as paramount or highly crucial.	Small sample with patients from single demographic area
The goldilocks conundrum: Disclosing discrimination risks in informed consent [51]	The study assessed how variant consent phrasings might alter an individual's viewpoints.	The inclination to participate was observed to shift based on variations in the language of informed consent. In cases where there was expansive detailing about anti-discrimination legislative protections, there was a noted decrease in participation enthusiasm.	Strength of change not measured.
Video education about genetic privacy and patient perspectives about sharing prenatal genetic data: a randomized trial [52]	Study investigated whether patients undergoing genetic testing were aware that their data might be shared for research and if they were willing to share their data.	A majority of the study's participants hadn't realized that their prenatal genetic information could be purposed for non-diagnostic uses. Those pregnant individuals who were informed about the Genetic Information Nondiscrimination Act didn't exhibit a greater willingness to disseminate their genetic information compared to those who weren't acquainted with the act.	Few numbers of patients deemed eligible.
The effects of a genetic information leaflet on public attitudes towards genetic testing [53]	To gauge the influence of a seemingly unbiased informational pamphlet on perceptions about genetic testing.	Recipients of this pamphlet manifested heightened comprehension levels about genetic testing and showcased amplified interest and favorable stances on genetic matters.	

## Conclusions

BPD arises from a complex interplay of genetic factors accounting for 40-60% of variation, and environmental influences, notably childhood trauma. Ethnicity has also been found to play a role in BPD susceptibility, marking it as one of the most inheritable psychiatric disorders. Various psychotherapy methods such as DBT, MBT, TFP, and schema therapy have shown effectiveness in BPD treatment, with genetic factors potentially influencing these outcomes. Emerging genetic testing approaches promise personalized treatment plans; however, they also pose ethical dilemmas, including privacy and stigmatization concerns. The optimization of treatment efficacy may be achieved through a blend of psychotherapy and pharmacotherapy, guided by genetic variations, despite the accompanying complexities and ethical challenges.

While our current understanding is limited due to factors such as recall bias, small sample sizes, and BPD's multifaceted nature, future research can pave the way toward a deeper comprehension of gene-environment interactions and the influence of additional genetic markers on BPD. Longitudinal studies with larger, more representative sample sizes are essential, alongside a focus on gender representation due to BPD's higher prevalence in females. With strategic planning, genetic testing has the potential to revolutionize BPD treatment, spearheading a future where therapy is personalized, early interventions are possible, and patient outcomes are considerably improved.
